# Clinical and pathological predictors of relapse in IgG4-related disease

**DOI:** 10.1186/s13075-022-02792-z

**Published:** 2022-05-11

**Authors:** Ji Zongfei, Chen Lingli, Sun Ying, Ma Lingying, Zhang Lijuan, Liu Dongmei, Dai Xiaomin, Hou Yingyong, Chen Huiyong, Ma Lili, Jiang Lindi

**Affiliations:** 1 Department of Rheumatology, Zhongshan Hospital, Fudan University, 180 Fenglin Road, Shanghai, China; 2 Department of Pathology, Zhongshan Hospital, Fudan University, 180 Fenglin Road, Shanghai, China; 3 Department of Rheumatology, Xiamen Branch, Zhongshan Hospital, Fudan University, 668 Jinhu Road, Xiamen, Fujian Province China; 4 Center of Clinical Epidemiology and Evidence-based Medicine, Fudan University, Shanghai, China

**Keywords:** IgG4-related disease, Relapse, Prognostic factor

## Abstract

**Objectives:**

In IgG4-related disease, the relationship between pathological findings and relapse has not been well established. This study aimed to identify the clinical and pathological predictors of disease relapse in IgG4-RD.

**Methods:**

Patients with newly diagnosed IgG4-RD (*n* = 71) were enrolled between January 2011 and April 2020; all cases were pathologically confirmed. The clinical and pathological features were recorded in a database at baseline and each follow-up visit. Patients were followed up at least once a month via outpatient clinic examinations and telephone calls. Univariate and multivariate Cox regression analyses and receiver operating curve (ROC) analysis were used to identify the predictors of disease relapse and to assess their predictive value.

**Results:**

Over a median follow-up of 26 (range, 6–123) months, 3/71 (4.2%) patients died. Of the remaining 68 patients, 47 (69.1%) patients had achieved clinical remission and 21 (30.9%) had suffered relapse at the last follow-up. The independent predictors of relapse were IgG4 ≥ 6.5 g/L (*HR* = 2.84, 95% *CI*: 1.11–7.23), IgG ≥ 20.8 g/L (*HR* = 4.11, 95% *CI*: 1.53–11.06), IgG4-RD responder index (RI) ≥ 9 (*HR* = 3.82, 95% *CI*: 1.28–11.37), and severe IgG4^+^ plasma cell infiltration (*HR* = 6.32, 95% *CI*: 1.79–22.41). A prognostic score developed using three of the identified predictors (IgG ≥ 20.8 g/L, IgG4-RD RI ≥ 9, and severe IgG4^+^ plasma cell infiltration) showed good value for predicting impending relapse (*AUC*, 0.806).

**Conclusions:**

In patients with IgG4-RD, IgG4 ≥ 6.5 g/L, IgG ≥ 20.8 g/L, IgG4-RD responder index (RI) ≥ 9, and severe IgG4^+^ plasma cell infiltration are predictors of relapse.

**Supplementary Information:**

The online version contains supplementary material available at 10.1186/s13075-022-02792-z.

## Key messages

- Among 68 patients with IgG4-RD, 21 (30.9%) suffered relapse during continuous treatment.
- Independent predictors of relapse were high IgG4, IgG, and IgG4-RD RI and severe IgG4^+^ plasma cell infiltration.
- A prognostic score developed using three of the identified predictors showed good predictive value.

## Introduction

IgG4-related disease (IgG4-RD) is a newly recognized systemic autoimmune disease that can involve the pancreatobiliary tract, retroperitoneum/aorta, head and neck region, and salivary glands [1]. The etiology is unknown. Single or multiple organomegaly may be present and, therefore, the disease often has to be differentiated from tumor [1]. Histopathological examination is one of the major criteria for the diagnosis of IgG4-RD according to the 2019 ACR/EULAR classification criteria, the characteristic findings being IgG4^+^ plasma cell infiltration, storiform fibrosis, and obliterative phlebitis [2, 3].

Relapse is common in IgG4-RD, occurring in 24–54% of patients after reduction of glucocorticoid dose [4–9]. The 2-year relapse rates are 23.0–41.4% [9, 10]. Patients with relapse present with reappearance of organomegaly and organ dysfunction (e.g., renal insufficiency, liver cirrhosis, intestinal obstruction). Previous studies have identified high serum IgG4 level, multiple organ involvement, high IgG4-RD responder index (RI) score, high serum IgE, and high eosinophil count at baseline as predictors of long-term relapse [11, 12]. Increase in memory B cell count after corticosteroid treatment and persistence of T-follicular helper cells after rituximab are also reported to be associated with relapse [13, 14]. In addition, our previous study [8] found serum TNF-α level and IL-2R to be independent predictors of relapse.

Although the pathological diagnostic criteria for IgG4-RD have been established [15–21], the pathological features associated with disease relapse are not known. The aim of this study was to investigate the relationship between pathological manifestations and disease relapse in IgG4-RD and to identify the independent predictors of disease relapse.

## Material and methods

### Patients

A total of 71 consecutive patients with newly diagnosed IgG4-RD were enrolled for this prospective study between January 2011 and April 2020 at the Zhongshan Hospital of Fudan University, Shanghai, China. Some patients visited our department directly while others were referred to us from various departments such as gastroenterology, pneumology, otolaryngology, general surgery, and urology. The patients were first classified as probable or definite IgG4-RD using the 2011 Japanese Comprehensive Clinical Diagnostic Criteria [15]. Those identified as “probable” IgG4-RD underwent comprehensive imaging examination for confirmation of the diagnosis [22]. The final diagnosis was made by two rheumatologists. All enrolled patients underwent needle biopsy or lesion resection. Patients with concurrent carcinoma, infection, hematological disease, or other rheumatic diseases were excluded. Of the 71 patients, 66 (93%) satisfied the 2019 ACR/EULAR classification criteria for diagnosis of IgG4-RD [23].

Enrolled patients were followed up through outpatient visits and/or telephone calls every month until disease relapse, death, or the end of the study (November 2020). All patients completed at least 6 months of follow-up. Demographic data, clinical features, laboratory examination results, pathological findings, imaging data, medical treatments, and disease outcomes were recorded in a database at baseline and each visit.

This study was approved by the Ethics Committee in Zhongshan Hospital of Fudan University (B2013-115(3)) and conformed to the ethical guidelines of the 1975 Declaration of Helsinki. Written informed consent was obtained from all patients.

### Serum IgG4 detection

The immune scatter turbidity method was used to detect the serum IgG4 level. All tests were performed using a Siemens BN II automatic protein analyzer in our hospital laboratory, with a limit of 0.08 g/L.

### Imaging examination

For the first 3 years, all patients underwent salivary gland ultrasound once every 3 months, chest and abdominal computed tomography (CT) scan once every 6 months, and/or head, neck, and abdominal magnetic resonance imaging (MRI) once every 6 months. Additional imaging examinations were performed if there was suspicion of reactivation. After the first 3 years, ultrasound was performed once every 6 months and CT once a year.

### Pathological examination

A total of 75 tissue samples were collected from the 71 patients and sent to the pathology department of our hospital. Sections were stained with hematoxylin and eosin (HE), elastic tissue stains, and immunohistochemical stains (including for markers of IgG and IgG4). The immunohistochemical antibodies used were rabbit monoclonal anti-IgG4 antibody (EP440, Abcam) and rabbit polyclonal anti-IgG antibody (A0423, Dako). The numbers of IgG4^+^ and IgG^+^ plasma cells were determined by manually counting the cells in five high-power fields (HPF) with a magnification of 400× under an optic microscope and calculating the average; the IgG4^+^/IgG^+^ plasma cell ratio was then calculated [2]. Two pathologists independently evaluated all specimens; a third pathologist was consulted if there was any disagreement.

### Treatment

Treatment was started with a glucocorticoid (prednisone), with or without an immunosuppressor agent. The dosage of prednisone was 0.6–0.8 mg/kg/day for the first 4 weeks; the daily dose was then gradually reduced by 5 mg every 2 weeks in the first 3 months and by 5 mg every month during the remaining follow-up period. The maintenance dose was 0.1–0.2 mg/kg/day. Patients with risk of irreversible organ damage or signs of multiorgan involvement also received single-agent immunosuppressor therapy with cyclophosphamide (0.6/m^2^ per month), azathioprine (1–2 mg/kg/day), mycophenolate mofetil (500–750 mg twice a day), or rituximab (100–200 mg/m^2^/week for 3–4 cycles, followed by one cycle every 6 months).

### Disease assessment and definitions

1. Disease activity was monitored using the IgG4-RD responder index (RI) [24], measured at baseline and then every 3 months during follow-up. The IgG4-RD RI score is based on clinical features and laboratory and imaging findings [18]. An expert team of two rheumatologists was responsible for the follow-up and disease activity assessment.
2. Remission was defined as a decline in IgG4-RD RI score by 50% and reduction in glucocorticoid dose to less than 0.2 mg/kg/day without relapse throughout the follow-up period [7, 25–27].
3. Relapse was defined as progressive disease or recurrence of clinical symptoms or imaging findings after remission, with or without elevation of the serum IgG4 level [7, 25].

### Clinical and pathological predictors

The following potential predictors of relapse were investigated: age, sex, medical history, clinical characteristics, laboratory findings, and pathological features.

### Statistical analysis

Normally distributed continuous variables were summarized as means ± standard deviation and non-normally distributed variables as medians and interquartile ranges. Categorical variables were summarized as frequencies and percentages. The independent two-sample Student’s *t*-test, the Mann–Whitney *U* test, the Kruskal–Wallis test, and the chi-square test were used, as applicable, for comparison between groups. The optimal cut-off values were determined by the receiver operating curve (ROC) analysis, and the area under the curve (AUC) was used to assess the predictive value of identified factors and the combined factors. Based on the fact that heavier infiltration was observed in lacrimal and salivary glands and lymph nodes than in visceral organs [2, 28], we estimated the optimal cut-off values separately for head and neck organs and visceral organs. The factors associated with relapse (binomial variable) were identified using univariate COX regression, Kaplan–Meier curves, and multivariate Cox regression (forward, selection). Two-tailed *p* < 0.05 was considered statistically significant. A predictive score for disease relapse was established using a multivariate Cox proportional hazard model (backward, condition). Both the *p* value and the clinical importance were concerned when the predictors were incorporated into the model [29, 30]. Statistical analysis was performed using SPSS 22.0 (IBM Corp., Armonk, NY, USA).

## Results

### Patient characteristics

A total of 71 patients (male: female ratio, 3.18:1) were enrolled. Table 1 shows the characteristics of the patients and the laboratory test results. The median age at diagnosis was 57 (47–63) years. The median time from onset of symptoms to diagnosis was 7 (2–22) months. Among the 71 patients, 59.2% (42/71) had ≥2 organ involvement, with median serum IgG4 of 3.90 (1.44–10.19) g/L. The most frequent clinical manifestations (see Supplementary Table 1, Additional file 1) were eye-related (20/71, 28.2%), digestive system–related (16/71, 22.5%), and low back pain/lower limb edema/hypertension (14/71, 19.7%).

**Table 1:** Baseline characteristics of the patients, stratified by relapse status

	All patients (*n* = 68)	Patients in remission (*n* = 47)	Patients with relapse (*n* = 21)	*p*
Age (years)	57 (47–63)	57.5 (46.8–64.0)	55.5 (41.3–62.8)	0.660^*^
Sex (male)	51 (75)	35 (74)	16 (76)	0.880^**^
Time from onset of symptoms to diagnosis (months)	7 (2–23)	6 (1–20)	12 (5–27)	0.081^*^
Organ involvement				
≥2	40 (58.8)	24 (51.1)	16 (76.2)	0.045^**^
≥3	23 (33.8)	13 (27.7)	10 (47.6)	0.112^**^
Head and neck involvement	24 (35.3)	17 (36.2)	7 (33.3)	0.821^**^
Visceral organ involvement	46 (67.6)	32 (68.1)	14 (66.7)	0.908^**^
Allergy history	14 (23.3)	9 (21.95)	5 (26.3)	0.710^**^
IgG4-RD RI	9 (6–12)	6 (6–12)	9 (6–12)	0.038^*^
Serum IgG4 (g/L)	3.90 (1.44–10.19)	3.09 (1.12–7.26)	7.26 (2.68–18.85)	0.026^*^
E (%)	2.05 (0.50–4.25)	2.7 (1.1–4.8)	1.5 (0.4–2.2)	0.074^*^
ESR (mm/h)	41 (12–83)	41 (13–82)	42 (10–92)	0.783^*^
CRP (mg/L)	6.1 (0.8–31.5)	6.8 (0.73–28.0)	4.97 (0.55–50.2)	0.955^*^
Serum IgG (g/L)	16.7 (11.5–22.9)	14.4 (10.9–20.3)	19.9 (15.5–34.3)	0.011^*^
Serum IgE (IU/mL)	112 (24–294)	112 (22–260)	119 (25–313)	0.831^*^
C3 (g/L)	0.99 (0.80–1.18)	1.02 (0.81–1.21)	0.92 (0.72–1.15)	0.196^*^
C4 (g/L)	0.19 (0.13–0.26)	0.23 (0.15–0.27)	0.16 (0.07–0.21)	0.019^*^
TG (mmol/L)	1.12 (0.82–1.83)	1.15 (0.94–1.87)	0.90 (0.71–1.54)	0.046^*^
TC (mmol/L)	3.71 (3.38-4.52)	4.18 (3.56–4.91)	3.38 (2.82–3.72)	0.005^*^
LDL (mmol/L)	2.20 (1.73-2.64)	2.50 (1.98–2.99)	1.92 (1.61–2.27)	0.030^*^

Data are *n* (%) or median (interquartile range)

*IgG4-RD RI* IgG4-related disease responder index, *E* eosinophils, *PLT* platelet count, *ESR* erythrocyte sedimentation rate, *CRP* C-reactive protein, *TC* total cholesterol, *TG* triglycerides, *LDL* low-density lipoprotein

^*^Mann–Whitney *U* test, ^**^chi-square test

### Treatment and follow-up

Over a median follow-up of 26 (range, 6–123) months, 3/71 (4.2%) patients died; the causes of death were infection (at 2 months), disease progression (intestinal obstruction complicated with septic shock at 5 months), and cerebral infarction (at 8 years). Of the remaining 68 patients, 13 (19.1%) received continuous glucocorticoid monotherapy, and 55 (80.9%) received continuous glucocorticoid plus immunosuppressor therapy. By the end of follow-up, 21/68 (30.9%) patients had relapsed, with a median time to relapse of 10 (6–30) months; the remaining 47/68 (69.1%) patients had sustained remission during a median follow-up of 17 (7–45.5) months. There was no significant difference in the therapy between the relapse patients and the remission patients.

### Pathological findings

A total of 75 tissue samples were obtained from the 71 patients. While 67 patients received biopsy at only one site, 4 patients received both organ biopsy and lymph node biopsy. The 75 tissue samples (see Supplementary Table 2, Additional file 1) came from retroperitoneal tissue (20.0%), lacrimal gland/orbit (17.3%), salivary glands (13.3%), sinuses (8.0%), and so on. The typical pathological findings are shown in Supplementary Fig. 1 and Fig. 2, Additional file 2.

Marked lymphocyte and plasmacyte infiltration was seen in all samples. All samples had >10 IgG4^+^ plasma cells/HPF, with the median count being 50 (30–117) IgG4^+^ plasma cell/HPF. In 73.3% (55/75) of samples, the ratio of IgG4/IgG-positive cells was ≥40%; the median ratio was 40% (33–56%). The IgG4^+^ plasma cell infiltration was significantly more severe in samples from the head and neck region (e.g., lacrimal/salivary gland, paranasal sinus, and cervical lymph nodes) than in samples from the viscera (e.g., lung, pancreas, liver and gall bladder, retroperitoneum): 90 (43–200) IgG4^+^ plasma cells/HPF vs. 45 (20–79) IgG4^+^ plasma cells/HPF, respectively (*p* = 0.010). The number of IgG4^+^ plasma cells per high-power field for each organ is shown in Supplementary Table 2, Additional file 1.

Obliterative phlebitis and fibrosis were seen in 30.7% (23/75) and 78.7% (59/75) samples, respectively. Other pathological manifestations are shown in Supplementary Table 3, Additional file 1. For patients who had biopsy from two sites, only the pathological findings in tissue obtained from the organs were considered.

### Association between baseline clinical characteristics and disease relapse

Table 1 and Supplementary Table 4, Additional file 1 show the general characteristics and laboratory test results of the 21 patients with relapse and the 47 patients with sustained remission. Relapse patients had significantly higher prevalence of ≥2 organ involvement (*p* = 0.045), higher IgG4-RD RI (*p* = 0.038), higher serum IgG4 (*p* = 0.026), and higher serum IgG (*p* = 0.011) at baseline; moreover, they had significantly lower baseline serum triglyceride (*p* = 0.046), total cholesterol (*p* = 0.005), low-density lipoprotein (*p* = 0.030), and complement 4 (*p* = 0.019) levels.

### Relationship between pathological manifestations and disease relapse

Compared to patients achieving remission, patients with relapse had significantly more IgG4^+^ plasma cell infiltration (*p* = 0.013, Table 2), higher prevalence of severe IgG4^+^ plasma cell infiltration—i.e., ≥60/HPF in visceral organs or ≥200/HPF in head and neck organs (*p* = 0.002), and lower prevalence of lymphoid follicle formation (*p* = 0.048). However, the prevalence of other pathological characteristics (see Table 2) was similar in relapse and remission patients.

**Table 2:** Comparison of pathological manifestations between the patients in remission and patients with relapse

	Patients in remission (*n* = 47)	Patients with relapse (*n* = 21)	*p*
Number of IgG4^+^ plasma cells/HPF	50 (25–120)	70 (40–290)	0.013^*^
Ratio of IgG4^+^ cell/IgG^+^ cell (%)	40.0 (30.0–56.0)	40.7 (40.0–59.5)	0.644^*^
Severe IgG4^+^ plasma cell infiltration (≥60/HPF in visceral organs or ≥200/HPF in head and neck organs)	21 (44.7%)	18 (85.7%)	0.002^**^
Fibrosis	39 (83.0)	18 (85.7)	0.777^**^
Storiform fibrosis	7 (14.9)	2 (9.5)	0.546^**^
Obliterative phlebitis	16 (34.0)	7 (33.3)	0.954^**^
Eosinophilic infiltration	5 (10.6)	1 (4.8)	0.430^**^
Lymphoid follicle formation	18 (38.3)	3 (14.3)	0.048^**^
Germinal center formation	5 (10.6)	1 (4.8)	0.430^**^

Data are *n* (%) or median (interquartile range). Statistically significant *p* values are in bold font

*HPF* high-power field

^*^Mann–Whitney *U* test, ^**^chi-square test

### Potential risk factors for disease relapse

In univariate Cox regression (Supplementary Table 5, Additional file 1), IgG ≥ 20.8 g/L (*HR* = 4.58, 95% *CI*: 1.40–15.01), IgG4 ≥ 6.5 g/L (*HR* = 3.28, 95% *CI*: 1.12–9.65), IgG4-RD RI ≥ 9 (*HR* = 3.75, 95% *CI*: 1.16–12.09), total cholesterol < 3.56 mmol/L (*HR* = 7.50, 95% *CI*: 1.69–33.27), serum triglycerides < 0.97 mmol/L (*HR* = 6.11, 95% *CI*: 1.42–26.36), low-density lipoprotein < 2.09 mmol/L (*HR* = 5.06, 95% *CI*: 1.20–21.42), and severe IgG4^+^ plasma cell infiltration (*HR* = 7.43, 95% *CI*: 1.92–28.68) were all associated with disease relapse. Number of involved organs ≥ 2, C4 < 0.19 g/L, and lymphoid follicle formation were not associated with relapse.

### Independent risk factors and a predictive score for disease relapse

In multivariate Cox regression (Table 3), the factors independently associated with relapse were IgG4 ≥ 6.5 g/L (*HR* = 2.84, 95% *CI*: 1.11–7.23), IgG ≥ 20.8 g/L (*HR* = 4.11, 95% *CI*: 1.53–11.06), IgG4-RD RI ≥ 9 (*HR* = 3.82, 95% *CI*: 1.28–11.37), and severe IgG4^+^ plasma cell infiltration (*HR* = 6.32, 95% *CI*: 1.79–22.41). The Cox proportional hazard model identified three factors for incorporation into the predictive model: IgG4-RD RI ≥ 9 (*p* = 0.026), severe IgG4^+^ plasma cell infiltration (*p* = 0.007), and IgG ≥ 20.8g/L (*p* = 0.056). Though the predictor IgG was not statistically significant, it was also incorporated into the predictive model due to its importance in clinical practice [8, 31]. Upon addition of IgG to the other two predictors, the optimism corrected AUC (95% *CI*) of this predictive model increased from 0.736 (0.610–0.861) to 0.806 (0.695–0.917).

**Table 3:** Cox regression multivariate analysis showing factors independently associated with IgG4-RD relapse

	*HR*	95% *CI*	*p*
IgG ≥ 20.8 g/L	4.11	1.53–11.06	**0.005**
IgG4 ≥ 6.5 g/L	2.84	1.11–7.23	**0.029**
IgG4-RD RI ≥ 9	3.82	1.28–11.37	**0.016**
Severe IgG4^+^ plasma cell infiltration	6.32	1.79–22.41	**0.004**

*IgG4-RD* IgG4-related disease, *HR* hazard ratio, *IgG4-RD-RI* IgG4-related disease responder index

**Fig. 1:**
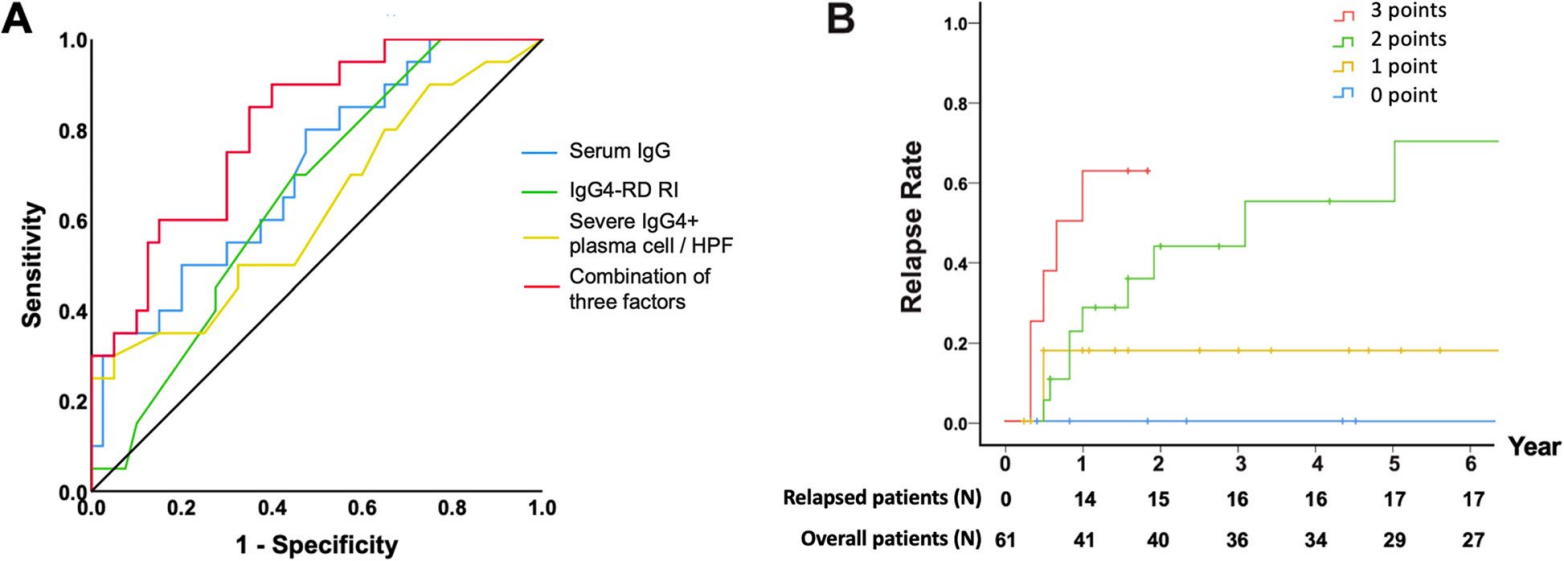
**A** ROC curves of the individual predictors and of the combination of predictors of disease relapse. **B** Cumulative relapse rates (Kaplan–Meier curve) of patients with different predictive scores

Figure 1A shows the ROCs of the individual predictors and of the combination of all three. The *AUC* (95% *CI*) for IgG, IgG4-RD RI, and severe IgG4^+^ plasma cell infiltration were 0.708 (0.572–0.845), 0.652 (0.514–0.791), and 0.712 (0.578–0.847), respectively. Thus, a predictive score was created by assigning 1 point for the presence of each of the three predictive factors; thus, the total score could range from 0 to 3. A score of ≥2 had sensitivity and specificity of 83% and 70%, respectively, for predicting the risk of relapse.

Figure 1B shows the cumulative relapse rate in patients with different predictive scores. The 1-year, 2-year, 3-year, and 5-year relapse rates were all significantly different between patients with different prognostic scores (Supplementary Table 6, Additional file 1). The prognostic score showed strong positive correlation with the 1-year, 2-year, 3-year, and 5-year relapse rates. The 3-year relapse rate for patients with 0, 1, 2, and 3 points were 0%, 27.3%, 63.6%, and 100%, respectively. The relationship between the three risk factors and disease relapse at 3 years is further illustrated by the matrix predictive model in Supplementary Table 7, Additional file 1.

## Discussion

The present study aimed to identify the clinical and pathological characteristics that predicted the risk of relapse in patients with IgG4-related disease. The independent predictive factors of disease relapse included IgG4 ≥ 6.5 g/L, IgG ≥ 20.8 g/L, IgG4-RD RI ≥ 9, and severe IgG4^+^ plasma cell infiltration. A prognostic score created using three of these risk factors (IgG ≥ 20.8 g/L, IgG4-RD RI ≥ 9, and severe IgG4^+^ plasma cell infiltration) showed good value for predicting the risk of relapse.

High IgG4-RD RI score and high IgG4 level have been found to be associated with relapse in previous studies also [12, 32, 33]. In a cohort of 52 patients with IgG4-RD, Culver et al. found that serum IgG4 ≥2.8 g/L at diagnosis predicted a risk of relapse [32]; however, the authors did not describe how the cut-off value was determined. In our study, ROC analysis showed the optimal cut-off value of serum IgG4 to be 6.5 g/L. It should be noted, however, that this cut-off is based on data from a single center. Thus, the relationship between serum IgG4 level and relapse needs further study.

Recent studies have investigated whether the 2019 ACR/EULAR classification criteria [34] and the novel clinical phenotypes [35] could predict the relapse of IgG4-RD. However, neither the classification score nor having a specific clinical phenotype of IgG4-RD seem to be associated with the risk of relapse. Further research is needed.

Previous studies on IgG4-RD have found that the degree of infiltration by IgG4-positive plasma cells differs between organs [2, 28], with heavier infiltration observed in lymph nodes, lacrimal and salivary glands, and the skin than in the pancreas, lungs, bile ducts, kidneys, aorta, and retroperitoneum [2]. This is consistent with our study, where the number of IgG4^+^ plasma cells per high-power field was significantly higher in tissues from the head and neck than from the viscera (pancreas, liver-gall bladder, and retroperitoneal tissues).

Marked IgG4^+^ plasma cell infiltration is one of the characteristic pathological manifestations of IgG4-RD. The pathogenesis of IgG4-RD remains unknown. Th2 cytokines (IL-4, IL-5, and IL-13) production can lead to IgG4 hyperproduction in B cell. IL-21-secreting Tfh cells could help germinal center formation and promote the differentiation from B cell to plasma cell. Several studies have demonstrated that the number of plasmablasts is positively correlated with disease activity and that the appearance of distinct plasmablast clones is associated with relapse of IgG4-RD [36, 37]. However, the relationship between the severity of IgG4^+^ plasma cell infiltration and disease activity or relapse is not well established.

In this study, severe IgG4^+^ plasma cell infiltration was found to be independently associated with disease relapse. In clinical practice, based on the findings of previous studies, patients with IgG4-RD are considered to have a low risk of relapse if they have single-organ involvement, low serum IgG4 level, and low IgG4-RD RI. However, our results suggest that those with a large number of IgG4^+^ plasma cells in the biopsied organ should also be considered to be at risk.

The prognostic score that we created using three predictive factors (IgG ≥ 20.8 g/L, IgG4-RD RI ≥ 9, and severe IgG4^+^ plasma cell infiltration) shows promise for predicting the risk of relapse. Therefore, we suggest that, for patients with a prognostic score of 2 or 3, maintenance treatment including steroid-sparing drugs is required. Meanwhile, patients with a score of 1 or 0 only need the initial course of steroids, and long-term treatment can probably be avoided.

The IgG4-RD RI score comprises the sum of organ sites’ scores plus the serum IgG4 concentration score [24]. Thus, the three risk factors that we selected for our scoring system take into consideration almost all the independent predictive factors identified in our study (i.e., IgG and IgG4 levels, organ involvements, and the severity of IgG4^+^ plasma cell infiltration). The elevation of total IgG might be due to elevated IgG1 [31] and/or IgG4 levels. Further studies are needed to confirm whether elevated IgG level has predictive value in all patients with IgG4-RD.

Zhang et al. classified IgG4-RD into two types according to the clinicopathological characteristics: a proliferative type and a fibrotic type [10]. Some features of the proliferative type are consistent with the predictive factors identified in our study, i.e., multiorgan involvement, severe IgG4^+^ plasma cell infiltration, and high serum IgG4 level [38, 39]. The proliferative type is likely to show a better response to therapy, but there is still no definite clinical evidence. The relationship between clinicopathological types and disease prognosis needs further study.

In the present study, different from our previous research [8], all patients had pathologically confirmed IgG4-RD. Our focus was on the relationship between the pathological features and relapse; we did not consider the prognostic value of uncommonly evaluated laboratory parameters (e.g., serum cytokine level).

Some limitations of this study must be acknowledged. The sample size was small, and all patients were from Shanghai or its peripheries in east China. However, a sample size of 71 was more than the calculated sample size of 63 for this diagnostic test (if sensitivity and specificity were both 0.8 ± 0.1). In addition, a selection bias may also exist because patients who had not received biopsy or resection were excluded. Therefore, whether our findings are applicable to all patients with IgG4-RD needs to be clarified in future research. In this study, we used the 2012 version of IgG4-RD RI and not the modified IgG4-RD RI published in 2018 [40]; this might also have affected our results.

## Conclusions

In patients with IgG4-RD, IgG4 ≥ 6.5 g/L, IgG ≥ 20.8 g/L, IgG4-RD responder index (RI) ≥ 9, and severe IgG4^+^ plasma cell infiltration appear to be independent predictors of relapse. The prognostic score created using three of the identified predictors (IgG, IgG4-RD RI, and severe IgG4^+^ plasma cell infiltration) could be a useful clinical tool to identify patients at high risk of relapse.

## Supplementary Information

**Additional file 1: Supplementary Table 1.** Demographic and clinical features of the patients (*n* = 71). **Supplementary Table 2.** Summary of the 75 tissue samples and the number of IgG4^+^ plasma cells per high-power field for each organ. **Supplementary Table 3.** Pathological features in the 75 tissue samples. **Supplementary Table 4.** Baseline patients’ characteristics stratified by relapsed status. **Supplementary Table 5.** The hazards ratio (HR) and diagnostic efficiencies of factors associated with IgG4-RD relapse by univariate analysis. **Supplementary Table 6.** The relapse rates in the patients with different prognostic score at 1, 2, 3, and 5 years. **Supplementary Table 7.** Relationship between the three risk factors and relapse at 3 years.

**Additional file 2: Supplementary Figure 1.** A. Lymphocyte and plasma cell (△) infiltration (H&E, ×400); B. storiform fibrosis (H&E, ×100) ; C. IgG4^+^ plasma cell (immumohistochemical staining, ×100); D. eosinophilia infiltration (→, H&E, ×400); E & F. obliterating phlebitis (H&E and elastic tissue stain, respectively, ×100). **Supplementary Figure 2.** A-D. Retroperitoneum tissue (×100): A. (H&E) Marked lymphocyte and plasma cell (→) infiltration and fibrosis (△); B. (H&E) lymphoid follicle formation (→); C & D. serial sections of IgG^+^ cell and IgG4^+^ cell (immunohistochemical staining). E-H. Submandibular gland (×100): : E. atrophic glandular acini and glandular ducts (circle) with marked lymphocyte and plasmacyte infiltration and fibrosis (H&E); F. genetic centre formation (H&E); G & H. serial sections of IgG^+^ cell and IgG4^+^ cell (immunohistochemical staining). Glomerular sclerosis (circle) with lymphocyte and plasmacyte infiltration (renal tissue); J. lymphocyte and plasma cell scattered within fibrosis; K. typical storiform fibrosis. I-K (H&E, ×100).

## Data Availability

The datasets generated during the current study are available from the corresponding author on reasonable request.

## Abbreviations

HPF: High-power field
HR: Hazard ratio
IgG4-RD RI: IgG4-related disease responder index
HB: Hemoglobin
WBC: White blood cell
E: Eosinophils
PLT: Platelet
ESR: Erythrocyte sedimentation rate
CRP: C-reactive protein
ALT: Alanine aminotransferase
AST: Aspartate aminotransferase
Cr: Serum creatinine
TC: Total cholesterol
TG: Triglycerides
LDL: Low-density lipoprotein
ROC: Receiver operating curve
AUC: The area under curve
