# Supplementary material for: Clinical and pathological predictors of relapse in IgG4-related disease
Source: Arthritis Res Ther. 2022 May 11;24:106. doi: 10.1186/s13075-022-02792-z (PMC9092827; doi:10.1186/s13075-022-02792-z)
Supplement: Supplementary file 1 — Additional file 1: Supplementary Table 1. Demographic and clinical features of the patients (n = 71). Supplementary Table 2. Summary of the 75 tissue samples and the number of IgG4+ plasma cells per high-power field for each organ. Supplementary Table 3. Pathological features in the 75 tissue samples. Supplementary Table 4. Baseline patients’ characteristics stratified by relapsed status. Supplementary Table 5. The hazards ratio (HR) and diagnostic efficiencies of factors associated with IgG4-RD relapse by univariate analysis. Supplementary Table 6. The relapse rates in the patients with different prognostic score at 1, 2, 3, and 5 years. Supplementary Table 7. Relationship between the three risk factors and relapse at 3 years. [file 13075_2022_2792_MOESM1_ESM.docx]

**Supplementary Table 1.** Demographic and clinical features of the patients (n = 71)

| **Symptoms and signs** | IgG4-RD |
| --- | --- |
| Digestive system | 16 (22.5%) |
| Low back pain/ lower limb edema/hypertension | 14 (19.7%) |
| Eye manifestations | 20 (28.2%) |
| Submandibular / neck mass | 8 (11.3%) |
| Ear, nose, and throat manifestation | 6 (8.5%) |
| Respiratory system | 3 (4.2%) |
| Skin symptoms | 1 (1.4%) |
| Systemic symptoms | 5 (7.0%) |
| Discovery of a lesion during medical checkup | 5 (7.0%) |

Data are n (%)

**Supplementary Table 2.** Summary of the 75 tissue samples and the number of IgG4^+^ plasma cells per high-power field for each organ

| Tissue | n (%) | number of IgG4^+^ plasma cells /HPF |
| --- | --- | --- |
| Retroperitoneum /Mediastinum | 16 (21.3%) | 43 (20-79) |
| Lacrimal gland/orbit | 13 (17.3%) | 100 (45-200) |
| Salivary glands | 10 (13.3%) | 100 (61-200) |
| Paranasal sinus | 6 (8.0%) | 45 (20-69) |
| Cervical lymph nodes | 5 (6.7%) | 62 (52-109) |
| Lung/[pleura](file:///C:/Users/flora/AppData/Local/Yodao/DeskDict/frame/20160210135958/javascript:void(0);) | 5 (6.7%) | 20 (10-180) |
| Pancreas | 4 (5.3%) | 40 (15-65) |
| Kidney/ureter | 4 (5.3%) | 138 (55-217) |
| Intestine/mesentery | 3 (4.0%) | 40 |
| Mastoid process | 3 (4.0%) | 66 |
| Liver | 2 (2.7%) | 33 |
| Gall bladder/biliary tract | 2 (2.7%) | 45 |
| Bone marrow | 1 (1.3%) | 10 |
| Larynx | 1 (1.3%) | 100 |

Data are n (%)

HPF, high-power field

**Supplementary Table 3**. Pathological features in the 75 tissue samples

| Tissue (N=75) | n (%) |
| --- | --- |
| Obliterative Phlebitis | 23 (30.7%) |
| Fibrosis | 59 (78.7%) |
| Lymphoid Follicles | 23 (30.7%) |
| Germinal Centre Formation | 6 (8%) |
| Eosinophil Infiltration | 6 (8%) |
| Neutrophil Infiltration | 1 (1.3%) |
| Fat necrosis | 1 (1.3%) |

**Supplementary Table 4**. Baseline patients’ characteristics stratified by relapsed status

|  | All patients (n = 68) | Remission patients  (n = 47) | Relapsed patients  (n = 21) | *p* |
| --- | --- | --- | --- | --- |
| Smoking history | 26 (38.2) | 20 (42.6) | 6 (28.6) | 0.273^**^ |
| Drinking history | 11 (16.7) | 8 (19.5) | 3 (15.8) | 0.729^**^ |
| Comorbidities  hypertension | 13 (19.1) | 8 (17.0) | 5 (23.8) | 0.511^**^ |
| hyperlipidaemia | 16 (28.6) | 11 (29.7) | 5 (26.3) | 0.789^**^ |
| HB (g/L) | 123 (102-133) | 124 (103-133) | 120 (100-132) | 0.582^*^ |
| WBC (×10^9^/L) | 6.42 (5.27-8.46) | 6.79 (5.41-9.35) | 6.32 (5.12-8.12) | 0.375^*^ |
| PLT (×10^9^/L) | 243 (186-312) | 248 (181-317) | 238 (213-306) | 0.929^*^ |
| Albumin (g/L) | 38 (36-40) | 38 (36-40) | 37.5 (31-39.8) | 0.391^*^ |
| Globulin (g/L) | 34 (27-42) | 32 (27-40) | 39 (26.5-51.5) | 0.101^*^ |
| ALT (U/L) | 13 (8-27) | 12.5 (8.8-29) | 13 (7-20) | 0.383^*^ |
| AST (U/L) | 16 (13-21) | 15.5 (13-21.5) | 16 (13-22) | 0.844^*^ |
| Cr (µmol/L) | 71 (61-89) | 74 (64-91) | 67 (59-83.5) | 0.405^*^ |
| Uric acid (µmol/L) | 324 (270-385) | 304 (264-384) | 331 (295-392) | 0.153^*^ |

Data are n (%) or median (interquartile range)

^*^Mann–Whitney *U* test, ^**^chi-square test

HB, haemoglobin; WBC, white blood cell count; E, eosinophils; PLT, platelet count; ALT, alanine aminotransferase; AST, aspartate aminotransferase; Cr, serum creatinine

**Supplementary Table 5**. The hazards ratio (HR) and diagnostic efficiencies of factors associated with IgG4-RD relapse by univariate analysis

|  | AUC | Sensitivity (%) | Specificity (%) | | HR | 95% CI | | *p* | | |
| --- | --- | --- | --- | --- | --- | --- | --- | --- | --- | --- |
| Number of involved organs ≥2 | 0.643 | 0.48 | 0.72 | | 3.07 | 0.97-9.74 | | 0.057 | | |
| IgG ≥20.8 g/L | 0.703 | 0.50 | 0.81 | | 4.58 | 1.40-15.01 | | **0.012** | | |
| IgG4 ≥6.5 g/L | 0.671 | 0.62 | 0.71 | | 3.28 | 1.12-9.65 | | **0.031** | | |
| IgG4-RD RI ≥9 | 0.655 | 0.71 | 0.56 | | 3.75 | 1.16-12.09 | | **0.027** | | |
| TC <3.56 mmol/L | 0.781 | 0.77 | 0.70 | | 7.50 | 1.69-33.27 | | **0.008** | | |
| TG <0.97 mmol/L | 0.698 | 0.69 | 0.70 | | 6.11 | 1.42-26.36 | | **0.015** | | |
| LDL <2.09 mmol/L | 0.716 | 0.73 | 0.70 | | 5.06 | 1.20-21.42 | | **0.028** | | |
| C4 <0.19 g/L | 0.717 | 0.63 | 0.72 | | 3.46 | 0.94-12.74 | | 0.063 | | |
| Severe IgG4^+^ plasma cell infiltration (≥60/HPF in visceral organs, or ≥200/HPF in head and neck organs) | 0.621 | 0.86 | 0.55 | | 7.43 | 1.92-28.68 | | **0.004** | |  |
| No lymphoid follicle formation | 0.620 | 0.38 | 0.86 | 3.72 | | | 0.96-14.46 | | 0.057 |  |

IgG4-RD RI, IgG4-related disease response index; AUC, area under the curve; HR, hazard ratio; CI, confidence interval; TC, total cholesterol; TG, triglycerides; LDL, low-density lipoprotein; HPF, high-power field

Statistically significant *p* values are in bold font.

**Supplementary Table 6**. The relapse rates in the patients with different prognostic score at 1, 2, 3, and 5 years. Cramer 's V represented the correlation between the prognostic score and relapse rates.

| Relapse rate (%) | | Prognostic score (number of risk factors) | | | | *p* (chi-square test) | Cramer 's V  (*p*) |
| --- | --- | --- | --- | --- | --- | --- | --- |
|  |  | 0 | 1 | 2 | 3 |  |  |
|  | 1 year  (n = 41) | 0% | 23.1% | 42.9% | 83.3% | 0.008 | 0.536 (0.008) |
|  | 2 year  (n = 40) | 0% | 25% | 50% | 100% | 0.001 | 0.622 (0.001) |
|  | 3 year  (n = 36) | 0% | 27.3% | 66.7% | 100% | 0.001 | 0.674 (0.001) |
|  | 5 year  (n = 29) | 0% | 42.9% | 81.8% | 100% | 0.002 | 0.724 (0.002) |

Red ≥70%, Green 50%-70%, Yellow 30%-49%

**Supplementary** **Table 7**. Relationship between the three risk factors and relapse at 3 years (matrix predictive model, n=36)

|  | IgG <20.8 g/L | IgG ≥20.8 g/L |  |
| --- | --- | --- | --- |
| Low IgG4^+^ plasma cell infiltration (<60/HPF in visceral organs, or <200/HPF in head and neck organs) | 0% | 33.3% | IgG4-RD RI <9 |
|  | 33.3% | 50% | IgG4-RD RI ≥9 |
| Severe IgG4^+^ plasma cell infiltration (≥60/HPF in visceral organs, or ≥200/HPF in head and neck organs) | 20% | 50% | IgG4-RD RI <9 |
|  | 75% | 100% | IgG4-RD RI ≥9 |

Red ≥70%, Green 50-70%, Yellow 30-49%
