# Supplementary material for: Clinical and pathological predictors of relapse in IgG4-related disease
Source: Arthritis Res Ther. 2022 May 11;24:106. doi: 10.1186/s13075-022-02792-z (PMC9092827; doi:10.1186/s13075-022-02792-z)
Supplement: Supplementary file 2 — Additional file 2: Supplementary Figure 1. A. Lymphocyte and plasma cell (△) infiltration (H&E, ×400); B. storiform fibrosis (H&E, ×100) ; C. IgG4+ plasma cell (immumohistochemical staining, ×100); D. eosinophilia infiltration (→, H&E, ×400); E & F. obliterating phlebitis (H&E and elastic tissue stain, respectively, ×100). Supplementary Figure 2. A-D. Retroperitoneum tissue (×100): A. (H&E) Marked lymphocyte and plasma cell (→) infiltration and fibrosis (△); B. (H&E) lymphoid follicle formation (→); C & D. serial sections of IgG+ cell and IgG4+ cell (immunohistochemical staining). E-H. Submandibular gland (×100): : E. atrophic glandular acini and glandular ducts (circle) with marked lymphocyte and plasmacyte infiltration and fibrosis (H&E); F. genetic centre formation (H&E); G & H. serial sections of IgG+ cell and IgG4+ cell (immunohistochemical staining). Glomerular sclerosis (circle) with lymphocyte and plasmacyte infiltration (renal tissue); J. lymphocyte and plasma cell scattered within fibrosis; K. typical storiform fibrosis. I-K (H&E, ×100). [file 13075_2022_2792_MOESM2_ESM.pptx]

## Slide 1
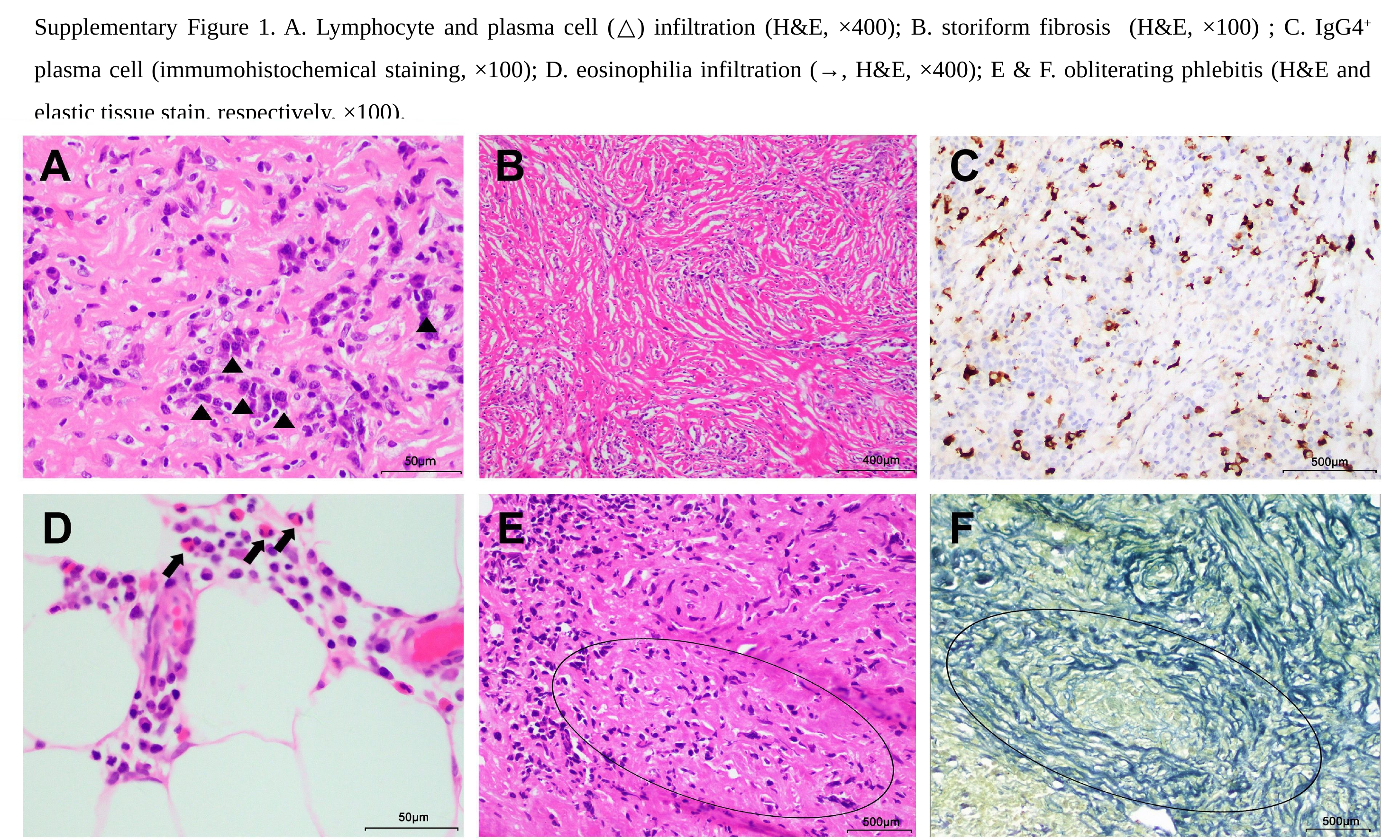

Supplementary Figure 1. A. Lymphocyte and plasma cell (△) infiltration (H&E, ×400); B. storiform fibrosis (H&E, ×100) ; C. IgG4+ plasma cell (immumohistochemical staining, ×100); D. eosinophilia infiltration (→, H&E, ×400); E & F. obliterating phlebitis (H&E and elastic tissue stain, respectively, ×100).
#

## Slide 2
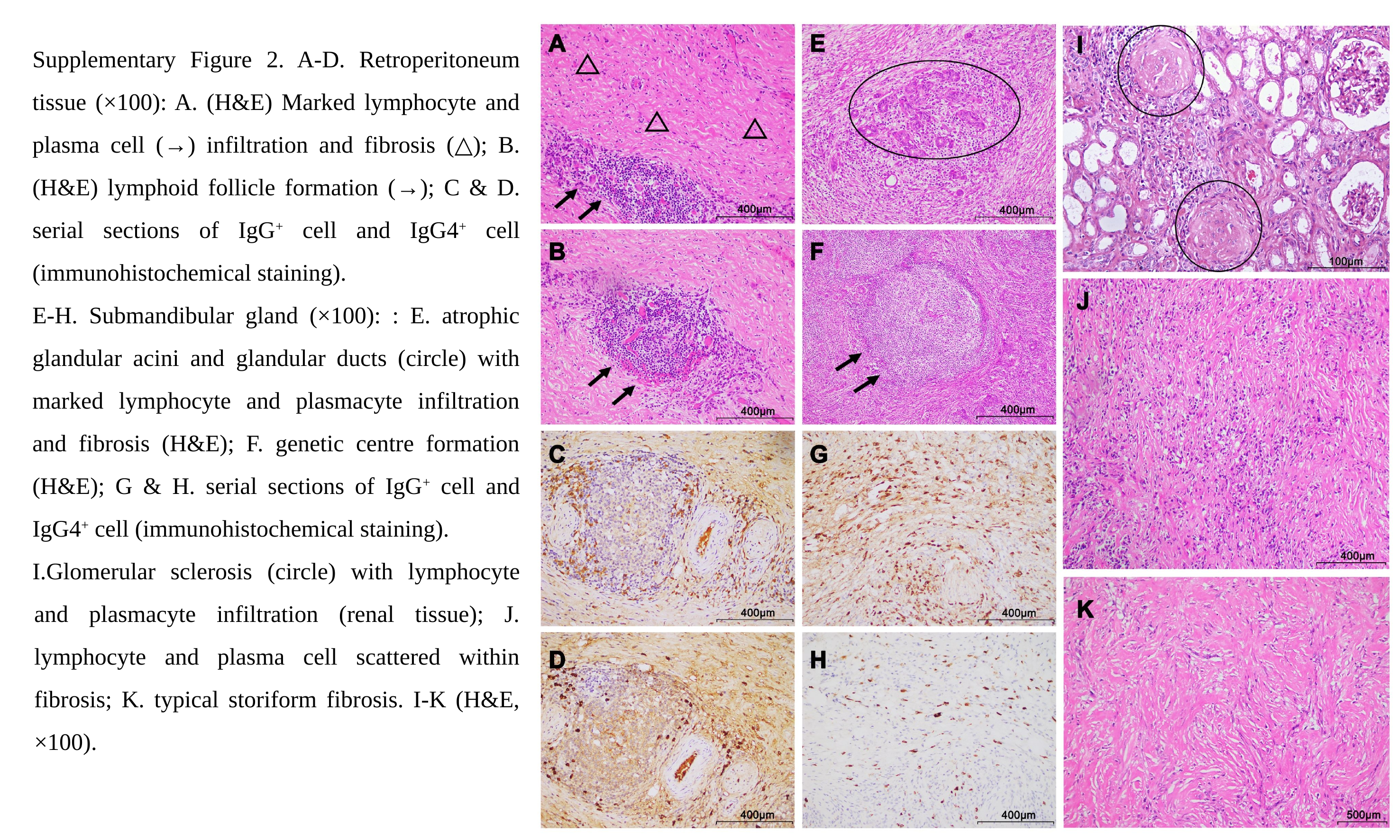

Supplementary Figure 2. A-D. Retroperitoneum tissue (×100): A. (H&E) Marked lymphocyte and plasma cell (→) infiltration and fibrosis (△); B. (H&E) lymphoid follicle formation (→); C & D. serial sections of IgG+ cell and IgG4+ cell (immunohistochemical staining).
E-H. Submandibular gland (×100): : E. atrophic glandular acini and glandular ducts (circle) with marked lymphocyte and plasmacyte infiltration and fibrosis (H&E); F. genetic centre formation (H&E); G & H. serial sections of IgG+ cell and IgG4+ cell (immunohistochemical staining).
Glomerular sclerosis (circle) with lymphocyte and plasmacyte infiltration (renal tissue); J. lymphocyte and plasma cell scattered within fibrosis; K. typical storiform fibrosis. I-K (H&E, ×100).
